# A preliminary study on the use of phycocyanin as a natural blue color source in toffee‐type soft candy: Effect of storage temperature and pigment concentration

**DOI:** 10.1002/fsn3.4401

**Published:** 2024-08-13

**Authors:** Recep Gunes, Ibrahim Palabiyik, Sefik Kurultay

**Affiliations:** ^1^ Department of Food Engineering, Faculty of Engineering Kirklareli University Kirklareli Türkiye; ^2^ Department of Food Engineering, Faculty of Agriculture Tekirdag Namik Kemal University Tekirdag Türkiye

**Keywords:** color, confectionery, phycocyanin, storage

## Abstract

In this study, phycocyanin obtained from *Spirulina platensis* extract was used at various concentrations as a natural blue color source in toffee‐type soft candy, and the effect of various storage temperatures (4, 20, and 40°C) on the color stability was investigated. In addition, main quality parameters, such as water activity, texture, and sensory analysis, were determined in the samples stored for 3 months at 20°C, and thus the effect of phycocyanin addition on the product appeal was studied. According to the results, the addition of phycocyanin powder did not significantly affect water activity (*p* > .05). As expected, L*, a*, and b* values of the samples changed with the phycocyanin, and the Δ*E* values of the samples stored at 4, 20, and 40°C varied between 0.14–0.44, 0.84–2.76, and 2.63–7.90, respectively, during the entire storage period. The texture analysis outputs revealed that the use of phycocyanin did not cause any change in the textural properties of the samples (*p* > .05). Considering the sensory analysis, all studied concentrations scored high in terms of color liking, and the use of phycocyanin in toffee‐type soft candy production resulted in a remarkable consumer appeal. Hence, the outcomes of this study show that if temperature control is performed in the production, the organoleptic properties of toffee‐type candy products can be enhanced using phycocyanin and can meet consumer requests and demands.

## Graphical abstract

Phycocyanin (PC) was used as a natural blue color source in toffee‐type soft candy. PC can be used successfully if the temperature is controlled during production. PC additive did not adversely affect the structural and physicochemical properties, and also there was no significant loss of PC stability in samples stored at 20°C.

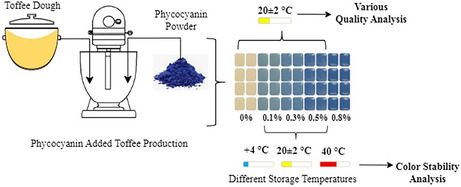

## 1 INTRODUCTION

Color is a key component to increase the ultimate appetizing value and consumer acceptance. In this regard, food coloring is used in both commercial food production and domestic cooking purposes (Dey & Nagababu, 2022). Today, synthetic food dyes (colorants) are generally preferred in many products manufactured in the food industry due to important properties, such as low cost, resistance to light, pH, temperature, and high color stability during storage. However, it is known that consuming these synthetic colorants may lead to severe health problems (Dey & Nagababu, 2022; Miller et al., 2022). In this regard, new trends in the food industry include the use of natural pigments derived from different sources with specific functions and health benefits, instead of artificial colorants (Altınok et al., 2020; Amjadi et al., 2018; Heidari et al., 2024; Hoang et al., 2024; Raei et al., 2017; Rodríguez‐Sánchez et al., 2017). However, despite the several natural sources, manufacturing food products containing only natural colorants is still a major challenge due to their chemical instability and degradation during food processing and storage (Neves et al., 2019).

Besides all this, among the natural colors, blue is probably the biggest challenge as its natural resources are limited. Also, there is no natural blue colorant with the same physical and chemical stability, coloring power, and easy scale‐up in production on the market yet, compared to the synthetics (Neves et al., 2021). In this context, *Spirulina platensis* (*Arthrospira platensis*), which appears characteristically blue–green due to the dominance of chlorophyll‐a (green) and phycocyanin pigment (blue), stands out as having significant potential in terms of different applications in the food industry (Muthulakshmi et al., 2012). As is known, phycocyanin (PC), which is found in high amounts in the biomass of *S. platensis*, is the most economically important phycobiliprotein type with a blue color. It is an odorless, nontoxic, water‐soluble pigment with high antioxidant and strong fluorescent properties (*λ*_max_ 615–640 nm), consisting of two relatively homologous subunits, *α* and *β*, and represents up to 20% of the dry mass of total cellular proteins (Bermejo et al., 2006; Chaiklahan et al., 2011). As a matter of fact, a significant part of the bioactivity exhibited by *S. platensis* is mainly associated with the significant antioxidant capacity of the phycocyanin compound (Stanic‐Vucinic et al., 2018). Today, the demand for phycocyanin, particularly as a natural blue colorant, has increased drastically after its approval by the Food and Drug Administration (FDA), and subsequently, its use in a variety of food products, such as ice cream and frozen desserts, edible toppings, powder form of different beverage mixes, dairy products, and various cereal‐based products, has been tried (Mysliwa‐Kurdziel & Solymosi, 2017; Sonani et al., 2016; Stanic‐Vucinic et al., 2018).

In the confectionery market, the class of aerated products covers a wide range of samples, from the highly aerated marshmallow to the lightly aerated (dense) toffee candy. As with the others, various synthetic colorants are used in terms of marketing strategy and visual appeal in toffee‐type products, which are in great interest and demand by all age groups (Gunes et al., 2022). Patent Blue (E 131), Indigo Carmine (E 132), and Brilliant Blue (E 133) synthetic colorants are most commonly used in these products in order to obtain different shades of “blue.” Although there are various fruit purees or fruit juices and herbal additives used in toffee‐type soft candy products in the literature, to the best of our knowledge, there is no study using phycocyanin as a natural blue color source. In this context, in the present study, phycocyanin in powder form was added to the toffee‐type soft candy samples at different concentrations and its effect on the quality parameters of the product was investigated. On the other hand, the stability of the phycocyanin in the samples was investigated by storing at different storage temperatures for 3 months.

## 2 MATERIALS AND METHODS

### 2.1 Materials

Phycocyanin powder (E18, moisture <8%, pH = 6), as a natural blue colorant, was obtained from Meilleur du Chef (Türkiye). Other ingredients used in the production of toffee‐type soft candy formulation were obtained from Smart Chemistry (Izmir/Türkiye).

### 2.2 Methods

#### 2.2.1 Toffee‐type soft candy production

The formulation of the toffee sample consists of water (15.00%), sugar (42.50%), sorbitol (3.00%), citric acid anhydrate (0.80%), glucose syrup (31.50%), glycerol monostearate (0.041%), tocopherol (0.032%), soy lecithin (0.007%), hydrogenated vegetable oil (5.00%), 250 bloom bovine gelatin (1.50%), salt (0.21%), and blueberry flavor (0.21%). In the production, first, a mixture of water, sugar, and glucose syrup (42 DE) was prepared and preheated at 100°C in a Thermomix (TM5, Vorwerk UK) at 200 rpm (revolutions per minute). Then, the temperature was increased to 120°C and hydrogenated vegetable oil, lecithin, sorbitol, salt, gelatin (Type A, 250 Bloom), and tocopherol were added. The heat treatment was continued by stirring at 200 rpm until the soluble solid content reached 90.0 ± 2.00°Brix. The cooking allowed for precise control of the final temperature and, as a result, standardization of the residual moisture content of the mass by about 5.5%–6.0%. After reaching the target‐soluble dry matter ratio, the mixture was cooled to 90°C and citric acid anhydrate was added and mixed well. The temperature of the mass was reduced to 50–55°C. In this step, flavoring (0.2 g/100 g liquid blueberry aroma) was added and the candy dough was rolled and kneaded for 20 min in a KitchenAid (USA) equipment. At the end of the period, the mass was transferred into a flat silicon mold, left at room temperature for 3–4 h, and cut in equal volumes (1.5 × 1.5 × 0.5 cm, 2.5 g) (Hartel et al., 2018).

#### 2.2.2 Addition of phycocyanin to the toffee‐type soft candy samples

Considering the general amount of colorants used in soft candy products, phycocyanin was added at the rate of 0.1%, 0.3%, 0.5%, and 0.8% (w/w) when the candy dough was rolled and kneaded (50–55°C) in a KitchenAid (USA) equipment. It was determined that the addition of 0.5% phycocyanin provided sufficient blueness taking into account the production cost, and therefore, the ratios of 0.1%, 0.3%, and 0.5% were kept constant in the production. All samples produced in this way were first wrapped in butter paper and then metallic‐coated polythene wraps. The samples were finally put in plastic bags (polyethylene, PE, 200G/50 μ) with a zipper (Chavan et al., 2015) and stored at 4 (±2), 20 (±2) and 40°C for 3 months.

#### 2.2.3 Water activity (a_w_) measurement

The water activity value of the samples stored at 20°C for 3 months was determined using a_w_ meter (Decagon AquaLab, 4 TE) in three replications on the 1st, 30th, 60th, and 90th days (AOAC, 2010; Samsiah et al., 2009).

#### 2.2.4 Color analysis

The color parameters of all samples stored at different storage temperatures were determined by using Chromameter CR‐400 Konica Minolta (Tokyo, Japan). The analysis was performed on the 1st, 30th, 60th, and 90th days. As a result of the analysis, L* (brightness), a* (redness–greenness), and b* (yellowness–blueness) values of all samples stored under different conditions were determined. Then, Δ*E* (color difference) values of the samples were calculated using these obtained values. According to the CIE Lab color system, the color difference between two colors is calculated according to Equation (1) (Pathare et al., 2013);

(1) $$\Delta E={\left[{\left(\Delta L*\right)}^{2}+{\left(\Delta a*\right)}^{2}+{\left(\Delta b*\right)}^{2}\right]}^{1/2}$$

#### 2.2.5 Instrumental texture analysis

The texture profile analysis (TPA) of all samples (1.5 × 1.5 × 0.5) produced within the scope of the study was detected by using a Texture Analyzer (TA.HDPlus, Stable Micro Systems) with cylindrical stainless steel probe P/2E. First, samples were taken into 3.5 mL containers and then were tested with a load cell of 50 kg, with a test speed of 5 mm s^−1^, a trigger force of 5 g (0.05 Newton, N), a depth of 3 mm, and a delay of 5 s between the first and second penetration. The total work applied was recorded using the force–distance curves. From these curves, the hardness (N), chewiness (N), cohesiveness, adhesiveness (N.sec), and resilience properties of the samples were determined. The analysis was carried out with at least five samples and was repeated on the 1st, 30th, 60th, and 90th days of storage at 20 ± 2°C for 3 months (Altınok et al., 2020; Nishinari et al., 2019; Utomo et al., 2014).

#### 2.2.6 Sensory analysis

Sensory evaluation of the samples stored at 20°C was carried out by a panelist group of 40 (20 females and 20 males, 22–36 years old) on the 1st, 30th, 60th, and 90th days during the 3‐month storage period. In the analysis, a 9‐point hedonic scale was used. According to the scale, 1 = dislike extremely, 9 = like extremely for color, taste, and general acceptability parameters; 1 = extremely soft, 9 = extremely hard for the hardness parameter, and 1 = not sticky, 9 = extremely sticky for the stickiness parameter (Shah et al., 2010). Prior to the evaluation, the panelists were informed about the composition of the samples. Then, the samples were coded with randomly selected numbers. Crackers and water were given between each evaluation during the analysis. The study was reviewed and approved by the Tekirdag Namik Kemal University and informed permission was acquired from each individual before their involvement in the study.

#### 2.2.7 Statistical analysis

The data obtained were analyzed statistically by using the Windows‐based SPSS 17.0.1 (SPSS Inc., Chicago, Illinois, USA) statistical package program, and one‐way analysis of variance (ANOVA) and Tukey's test were used to determine whether there was a statistical difference between the groups (*p* < .05). In addition, using the bivariate Pearson correlation test, it was evaluated whether there was a correlation between the sensorial and instrumental texture analysis results.

## 3 RESULTS AND DISCUSSION

### 3.1 Water activity (a_w_) results

It is very important to control and monitor the product moisture in order to produce high‐quality and long shelf life soft confectionery products (Cano‐Lamadrid et al., 2020; Hartel et al., 2018). However, moisture content alone may not be sufficient to fully characterize product quality. In this regard, water activity (a_w_) is a very important parameter frequently used to determine microbial stability, product texture, and moisture migration during storage (Ergun et al., 2010; Subramaniam, 2016). In this context, water activity (a_w_) results of the phycocyanin‐free (P0) and phycocyanin added (P0.1, P0.3, and P0.5) toffee‐type soft candy samples stored at 20°C are given in Table 1. In the literature, it has been stated that the a_w_ values of this type of candy product are generally between 0.45 and 0.60 according to softness or hardness state (Ergun et al., 2010). In our study, it was seen that the a_w_ values in all samples were within this specified value range. On the other hand, the highest a_w_ values were recorded in the samples belonging to the phycocyanin‐free group (*p* < .05). However, the increase in the concentration of phycocyanin did not cause a linear increase or vice versa in the a_w_ values of the samples. It was also determined that the a_w_ values of all samples decreased in parallel with the storage time (*p* < .05), but it should be noted that this decrease occurred independently of the presence and amount of phycocyanin.

**TABLE 1:** Water activity and moisture values of toffee‐type soft candy samples.

Sample	Water activity values			
	1st day	30th day	60th day	90th day
P0	0.5957 ± 0.0013^Aa^	0.5785 ± 0.0007^Ab^	0.5565 ± 0.0018^Ac^	0.5361 ± 0.0013^Ad^
P0.1	0.5812 ± 0.0016^Ca^	0.5613 ± 0.0010^Cb^	0.5388 ± 0.0013^Cc^	0.5278 ± 0.0009^Bd^
P0.3	0.5745 ± 0.0010^Da^	0.5573 ± 0.0014^Db^	0.5297 ± 0.0020^Dc^	0.5155 ± 0.0009^Dd^
P0.5	0.5886 ± 0.0020^Ba^	0.5689 ± 0.0016^Bb^	0.5428 ± 0.0017^Bc^	0.5242 ± 0.0014^Cd^

*Note*: Data represent average values ± standard deviation. There is no significant difference between the results shown with the same capital letter in the same column for the same storage period (*p* > .05). There is no significant difference between the results shown with the same lowercase letter in the same line for the same analysis results (*p* > .05). P: phycocyanin, 0, 0.1, 0.3, and 0.5: phycocyanin additive percentage.

Besides all this, in the present study, unlike commercial manufacturing processes, phycocyanin was added at the pulling step in the production to prevent it from being affected by the high temperature. From this point, the addition of a liquid component in the production during the pulling may cause undesirable changes in the a_w_ value of the product. In this sense, according to the results, the addition of phycocyanin in “powder” instead of liquid form is considered to be a very important output in terms of not having a negative effect on the initial a_w_ values of these types of products. To the best of our knowledge, there is no study yet on the use of phycocyanin in toffee‐type candy products. However, as an example of the use of phycocyanin as a colorant in a different candy product, Dewi et al. (2018) included microencapsulated phycocyanin in the production of jelly‐type candy at different concentrations (1%, 3%, and 5%) by diluting it with 100 mL of water. Considering the results of the study, the a_w_ values of the samples were determined as 0.869, 0.875, and 0.879, respectively. It was stated that the increase in a_w_ values was caused by the application of low temperature during the production due to the sensitivity of phycocyanin to heat and the inability to remove excess moisture from the candy mass depending on the amount of “liquefied” phycocyanin. Based on these results, it is obvious that optimization method should be made in terms of the use of phycocyanin in jelly‐type candy production. In another study, Da Silva, Annetta, et al. (2016) used the spray‐drying (SD) and freeze‐drying (FD) powders of açai (*Euterpe oleracea* Mart.) fruit in the manufacture of the same type of candy as in the present study. Fruit powders (5%, w/w) were included in the production during the pulling and cooling step (45–50°C) in order to benefit from both the coloring and functional properties of the powders, and the water activity values of the samples produced by adding SD and FD powders were found to be 0.47 and 0.49, respectively.

### 3.2 Color analysis results

**FIGURE 1:**
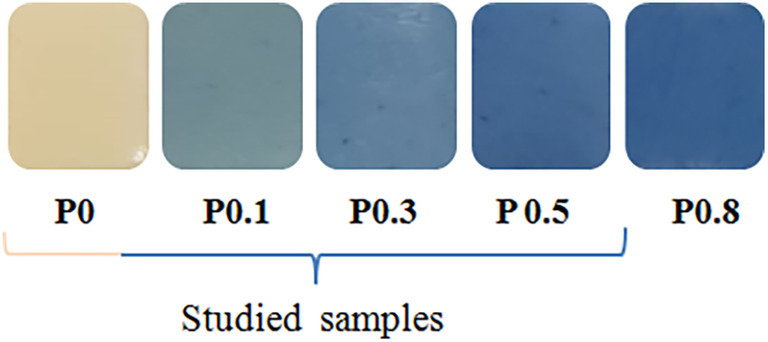
The surface views of all samples produced within the scope of the study. P, phycocyanin, 0, 0.1, 0.3, 0.5, and 0.8: phycocyanin additive percentage.

Color is one of the most important properties that determine the quality and desirability of food products. In this context, the surface views of all the samples produced are given in Figure 1, and the color properties of the samples on the first day are given in Table 2. As seen in Table 2, it was observed that there was a significant decrease in the L* values of the samples caused by the addition of phycocyanin, and this decrease was found to be directly proportional to the phycocyanin concentration (*p* < .05). The addition of phycocyanin changed the a* value of the uncolored sample (P0) and caused an increase in the greenness values (‐a*) of the samples. However, interestingly, in parallel with the increase in phycocyanin concentration, the same effect was not observed in the ‐a* values of the samples, and it was determined that the P0.3 sample had the highest value. As expected, the addition of phycocyanin increased the blueness (‐b*) values of the samples and it was found that this effect was related to the colorant concentration, as in the P0.5 sample with the highest value (*p* < .05). According to these results, one of the most important outputs of the present study is that phycocyanin, which has thermal sensitivity, has been successfully included in the production of toffee‐type soft candy without any loss in its blue color capacity due to the production process. As stated in the production method, phycocyanin was added at a relatively low temperature (50–55°C) during the rolling and kneading step of the candy dough after the cooking process, which prevented the degradation of phycocyanin due to temperature. As is known, there are some applications such as encapsulation for physical or chemical instability problems of natural compounds (Zabot et al., 2022), but such cost‐increasing additional treatments may not be needed if temperature control is carried out properly. Moreover, in different studies, sucrose and other small saccharides, as well as polyols, have been recognized as co‐solutes having an effective thermo‐protectant ability against phycocyanin denaturation (Antelo et al., 2008; Kannaujiya & Sinha, 2016; Martelli et al., 2014). Therefore, based on the results we obtained in the study, it can be stated that the sugar concentration in toffee‐type products has a positive effect on phycocyanin and provides an advantage in terms of maintaining the color stability of the pigment. This result shows that it would be beneficial to perform color stability studies depending on the amount and type of carbohydrate in the product matrix.

**TABLE 2:** The color characteristics of the samples on the first day.

Sample	L*	a*	b*
P0	83.41 ± 0.35	2.73 ± 0.10	19.39 ± 0.34
P0.1	63.13 ± 0.39^A^	−11.33 ± 0.11^B^	−8.09 ± 0.17^C^
P0.3	53.89 ± 0.66^B^	−13.15 ± 0.13^A^	−18.56 ± 0.22^B^
P0.5	48.65 ± 0.41^C^	−10.44 ± 0.10^C^	−23.19 ± 0.13^A^

*Note*: Data represent average values ±standard deviation. P: phycocyanin, 0, 0.1, 0.3, and 0.5: phycocyanin additive percentage (%). Statistical analysis was performed only among the phycocyanin‐added samples and there is no significant difference between the results shown with the same capital letter in the same column (*p* > .05).

**FIGURE 2:**
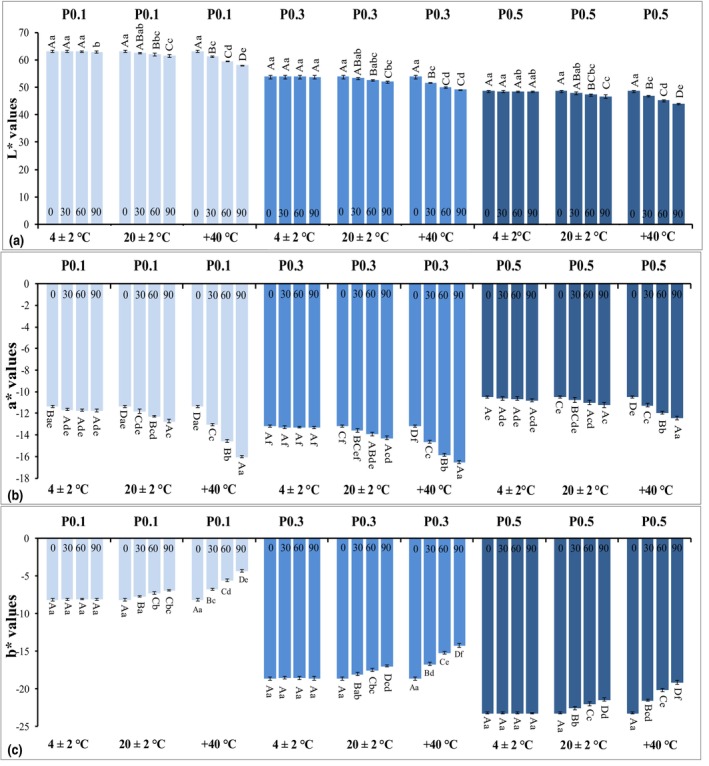
Time‐dependent change in color parameters of colored samples stored at different temperatures. (a) L* values, (b) a* values, (c) b* values. 0, 30, 60, and 90 refer to storage times. 4 ± 2°C, 20 ± 2°C, 40°C refers to storage temperatures. P, phycocyanin, 0.1, 0.3, and 0.5: phycocyanin percentage. There is no significant difference between the results shown with the same exponential capital letter for the same sample at the same storage temperature (*p* > .05). There is no significant difference between the results shown with the same exponential lowercase letter for the same sample with the same phycocyanin concentration in all storage temperatures (*p* > .05).

In addition to all these, it is very important to detect the stability of an active substance over time under the influence of various environmental factors, such as temperature, humidity, and light, to reveal the shelf life and recommended storage conditions. In this regard, the investigation of the color stability of the phycocyanin‐containing samples at different storage temperatures during 3 months constitutes an important part of the present study. According to this, the L*, a*, and b* values of the colored samples stored at 4, 20, and 40°C are given together in Figure 2, respectively.

According to Figure 2a, the greatest change showing itself as a decrease in L* values measured during the storage period was observed in the samples stored at 40°C in all three groups (*p* < .05). Within the scope of the study, there was no change in the L* values of the samples stored at 4°C (*p* > .05), while the application of storage at 20°C had a very slight but statistically significant effect on the L* values of the samples in all three groups during the entire storage period (*p* < .05). The most significant change in ‐a* values, which was determined as an increase depending on the storage period, was observed again in the samples stored at 40°C in all groups (Figure 2b). The remarkable result here is that the change in the ‐a* values of the samples with the highest phycocyanin concentration (P0.5) is less than the other groups depending on the storage time at 40°C. The same trend was also seen in ‐b* values and the increase in storage temperature caused a decrease in this parameter in all groups (Figure 2c).

Indeed, the time‐dependent change in samples kept at different storage temperatures compared to the initial sample can best be demonstrated with the Δ*E* value. As is known, the Δ*E* value is a measurement of the difference between two colors, and the higher Δ*E* value means the greater difference between the colors being compared. In the literature, differences in perceivable color are analytically classified as very significant Δ*E* > 3, significant 1.5 < Δ*E* < 3, and very small difference Δ*E* < 1.5 (Adekunte et al., 2010). In this context, calculated Δ*E* values of the colored samples stored at different temperatures depending on the storage time are given in Table 3. Considering the results, it was determined that the Δ*E* values of the samples stored at 4, 20, and 40°C varied between 0.14–0.44, 0.84–2.76, and 2.63–7.90, respectively, during the entire storage period. Depending on the increase in storage temperature, the highest Δ*E* values were observed in the samples stored at 40°C. Also, it was determined that there was an increase in the Δ*E* values of each storage application depending on the time. Based on these results, it is obvious that the storage temperature is important for the color characteristics of phycocyanin added toffee‐type soft candy products. However, these parameters are the results obtained with instrumental techniques, thus it should be monitored with sensorial evaluations and revealed whether the detected changes create noticeable results for consumers. On the other hand, from a practical perspective, it is stated that the average human eye cannot detect any color differences with a Δ*E* value of 3 or less (Chang et al., 2015). In this regard, considering that markets and shopping malls can control the temperatures by using air conditioners (Motoki et al., 2018), an ambient temperature of 20–25°C will not cause significant changes in the color characteristics of phycocyanin added toffee‐type products that remain on the market shelves for a certain period of time.

**TABLE 3:** Δ*E* values of the samples stored at different temperatures depending on time.

Sample/days	1st day	30th day	60th day	90th day
Δ*E* values of samples stored at 4°C				
P0.1	‐	0.28 ± 0.05	0.34 ± 0.11	0.40 ± 0.07
P0.3	‐	0.14 ± 0.05	0.14 ± 0.05	0.16 ± 0.05
P0.5	‐	0.20 ± 0.07	0.26 ± 0.10	0.44 ± 0.10
Δ*E* values of samples stored at 20 ± 2°C				
P0.1	‐	0.84 ± 0.12	1.65 ± 0.07	2.45 ± 0.11
P0.3	‐	0.93 ± 0.36	1.81 ± 0.34	2.72 ± 0.33
P0.5	‐	1.01 ± 0.14	1.90 ± 0.16	2.76 ± 0.23
Δ*E* values of samples stored at 40°C				
P0.1	‐	2.85 ± 0.85	5.51 ± 0.23	7.90 ± 0.24
P0.3	‐	3.25 ± 0.43	5.76 ± 0.46	7.28 ± 0.49
P0.5	‐	2.63 ± 0.23	4.86 ± 0.12	6.52 ± 0.22

### 3.3 Texture analysis results

In product development studies, it is of great importance to determine whether the textural properties of foods are in line with consumer demands. In this respect, determining the effects of any ingredient added to the formulation on the product texture is critical both in terms of the production process and marketing strategies. In Table 4, the hardness (N), chewiness (N), cohesiveness, adhesiveness (N.sec), and resilience parameters of the samples are given. According to the results, the hardness properties of the samples containing different amounts of phycocyanin were determined in the range of 15.0–15.3 N at the beginning of storage (*p* > .05), and it was found that the increase in the phycocyanin did not have any effect on the values. Based on various studies, the hardness value of such products is affected by many factors, including the production process, moisture content, various sweeteners, fats, and the type and amount of hydrocolloid used in the formulation, the air/foam content of the product, and the presence of different minor components (Čižauskaitė et al., 2019; Ergun et al., 2010; Figiel & Tajner‐Czopek, 2006; Hartel et al., 2018). For instance, according to the study of Da Silva, Queiroz, et al. (2016), it was determined that the hardness of the chewy candy depending on the polyol(s) used in the preparation varied from very soft (0.18–0.77 N) and soft (4.08–25.43 N) to hard (56.39 N) and very hard (171.09 N). In another study, Altınok et al. (2020) found that the hardness of soft candy samples containing grape seed and skin powder varied between 11.34–47.53 N and 16.79–24.08 N, respectively. In the meantime, the hardness values of the samples increased during the storage period, and changed between 35.596 N and 35.927 N at the end of the storage period (*p* > .05), similar to the study of Da Silva, Annetta, et al. (2016). As seen in Table 4, the same trend was also observed in chewiness values. Hardness and chewiness parameters are remarkable, especially in terms of the average age of the consumers. For instance, relatively low salivary flow rate, mouth volume, relatively few teeth, low tongue pressure, and decreased bite force are known to affect food preferences and expectations in elderly individuals (Ketel et al., 2020). Therefore, considering that different countries have a wide age range in terms of consumers, it is very important to adjust the textural properties of these products according to this scope and of course, preserve the structure during the retail on the market shelves. In this context, textural changes can be prevented by the use of different components in the production, especially shortenings, as well as by making better packaging.

**TABLE 4:** The textural properties of samples stored at 20°C for 3 months.

Sample	Parameter	1st day	30th day	60th day	90th day
P0	Hardness (*N*)	15.039 ± 0.246^Ad^	23.469 ± 0.745^Ac^	29.863 ± 0.673^Ab^	35.596 ± 0.854^Aa^
P0.1		15.130 ± 0.184^Ad^	24.309 ± 0.318^Ac^	30.109 ± 0.293^Ab^	35.778 ± 0.812^Aa^
P0.3		15.281 ± 0.487^Ad^	24.768 ± 1.067^Ac^	30.496 ± 0.263^Ab^	35.816 ± 0.787^Aa^
P0.5		15.064 ± 0.332^Ad^	24.017 ± 0.618^Ac^	30.217 ± 0.413^Ab^	35.927 ± 0.841^Aa^
P0	Chewiness (*N*)	1.514 ± 0.172^Ac^	2.207 ± 0.322b^Ab^	3.178 ± 0.138^Aa^	3.666 ± 0.153^Aa^
P0.1		1.539 ± 0.057^Ad^	2.260 ± 0.163^Ac^	3.308 ± 0.107^Ab^	3.619 ± 0.145^Aa^
P0.3		1.566 ± 0.144^Ac^	2.425 ± 0.209^Ab^	3.425 ± 0.149^Aa^	3.735 ± 0.101^Aa^
P0.5		1.476 ± 0.098^Ad^	2.137 ± 0.162^Ac^	3.293 ± 0.156^Ab^	3.786 ± 0.033^Aa^
P0	Cohesiveness	0.163 ± 0.004^Aa^	0.154 ± 0.005^Aa^	0.141 ± 0.002^Ab^	0.133 ± 0.005^Abc^
P0.1		0.166 ± 0.001^Aa^	0.156 ± 0.004^Aab^	0.145 ± 0.005^Ab^	0.134 ± 0.005^Ac^
P0.3		0.168 ± 0.005^Aa^	0.158 ± 0.002^Aab^	0.150 ± 0.001^Ab^	0.139 ± 0.005^Ac^
P0.5		0.165 ± 0.006^Aa^	0.151 ± 0.005^Aab^	0.143 ± 0.005^Ab^	0.136 ± 0.005^Abc^
P0	Adhesiveness (N.sec)	−0.106 ± 0.006^Aa^	−0.097 ± 0.005^Aab^	−0.089 ± 0.005^Ab^	−0.033 ± 0.006^Ac^
P0.1		−0.100 ± 0.002^Aa^	−0.096 ± 0.003^Aab^	−0.086 ± 0.001^Ab^	−0.032 ± 0.004^Ac^
P0.3		−0.103 ± 0.017^Aa^	−0.092 ± 0.002^Aab^	−0.085 ± 0.003^Ab^	−0.031 ± 0.002^Ac^
P0.5		−0.104 ± 0.010^Aa^	−0.095 ± 0.009^Aab^	−0.087 ± 0.003^Ab^	−0.031 ± 0.002^Ac^
P0	Resilience	0.025 ± 0.000^Aa^	0.024 ± 0.000^Aa^	0.023 ± 0.000^Abc^	0.022 ± 0.000^Ac^
P0.1		0.024 ± 0.000^Aa^	0.025 ± 0.000^ABa^	0.022 ± 0.001^Bb^	0.021 ± 0.001^Ab^
P0.3		0.024 ± 0.001^Aab^	0.025 ± 0.000^ABa^	0.022 ± 0.000^Bb^	0.022 ± 0.001^Ab^
P0.5		0.025 ± 0.001^Aa^	0.025 ± 0.001^Aa^	0.022 ± 0.000^Bb^	0.022 ± 0.001^Ab^

*Note*: Data represent average values ± standard deviation. There is no significant difference between the results shown with the same capital letter in the same column for the same parameter (*p* > .05). There is no significant difference between the results shown with the same lowercase letter in the same line (*p* > .05). P: phycocyanin, 0, 0.1, 0.3, and 0.5: phycocyanin additive percentage.

The indicator of the strength of the internal bonds of food is characterized as cohesiveness, while the work required to overcome the attractive forces between the surface of food and the surface of other materials with which the food comes into contact is defined as adhesiveness (Szczesniak, 2002). Considering the results of the first day, it was determined that the amount of phycocyanin used did not have a significant effect on the cohesiveness and adhesiveness values of the samples (*p* > .05). However, both cohesiveness and adhesiveness values decreased with storage time (*p* < .05). The decrease in adhesiveness values can be associated with the decrease in the water activity of the samples; on the other hand, there are studies stating that there exists a negative (Clarke & Farrell, 2000) or a positive up to a certain critical point (Tobin et al., 2017) or completely directly proportional (Al‐Muhtaseb et al., 2013; Zheng et al., 2016) relationship between the cohesiveness and moisture content of the product. Finally, the use of phycocyanin did not significantly affect the resilience values of the samples (*p* > .05) and negligible changes were determined in this parameter depending on the time during storage.

### 3.4 Sensory analysis results

The measurement of food acceptance is highly complex and relies on instrumental and sensorial determinations. In this regard, sensory evaluation tests are very important in determining whether the product is in line with the expectations (Świąder & Marczewska, 2021). Accordingly, in the present study, the sensory analysis results of all samples stored at 20°C for 3 months are given in Table 5. As seen in the table, the addition of different concentrations of phycocyanin was evaluated with statistically equivalent very high scores in terms of color liking compared to the control group (*p* > .05). In this case, it can be emphasized that each concentration of phycocyanin is highly appreciated and even the lowest concentration (P0.1) is remarkable in terms of color preference, considering the production cost. Moreover, it was noted that there was no change in the scores obtained in each evaluation period performed during the storage time (*p* > .05).

**TABLE 5:** The sensory analysis results of the samples stored at 20°C.

Parameter/day	P0	P0.1	P0.3	P0.5
Color/1	7.4 ± 0.7^Ab^	7.8 ± 0.9^Aab^	8.4 ± 0.9^Aa^	8.5 ± 0.5^Aa^
Color/30	7.1 ± 0.7^Ab^	8.1 ± 1.2^Aa^	8.5 ± 0.5^Aa^	8.8 ± 0.4^Aa^
Color/60	7.2 ± 0.6^Ab^	8.2 ± 0.6^Aa^	8.3 ± 0.6^Aa^	8.6 ± 0.5^Aa^
Color/90	6.8 ± 0.6^Ac^	7.8 ± 0.6^Ab^	8.1 ± 0.7^Aab^	8.7 ± 0.5^Aa^
Taste/1	8.4 ± 0.8^Aa^	8.1 ± 0.7^Aa^	8.4 ± 0.5^Aa^	8.3 ± 0.5^Aa^
Taste/30	8.0 ± 0.6^Aa^	8.1 ± 0.9^Aa^	8.3 ± 0.7^Aa^	8.2 ± 0.8^Aa^
Taste/60	8.1 ± 0.7^Aa^	8.4 ± 0.7^Aa^	8.6 ± 0.5^Aa^	8.5 ± 0.7^Aa^
Taste/90	7.9 ± 0.6^Aa^	8.3 ± 0.5^Aa^	8.4 ± 0.5^Aa^	8.4 ± 0.5^Aa^
Hardness/1	3.2 ± 0.9^Ca^	3.5 ± 0.5^Ba^	3.0 ± 0.5^Ca^	3.6 ± 0.7^Ca^
Hardness/30	4.0 ± 0.8^BCa^	4.1 ± 0.7^Ba^	4.2 ± 0.8^Ba^	4.2 ± 0.8^BCa^
Hardness/60	5.1 ± 0.7^Aa^	5.0 ± 0.7^Aa^	5.1 ± 0.7^Aa^	5.0 ± 0.8^ABa^
Hardness/90	4.8 ± 0.4^ABa^	4.9 ± 0.6^Aa^	4.8 ± 0.4^ABa^	5.2 ± 0.6^Aa^
Stickiness/1	4.7 ± 1.2^Aa^	4.3 ± 1.1^Aa^	4.5 ± 0.8^Aa^	4.9 ± 0.6^Aa^
Stickiness/30	3.0 ± 0.7^Ba^	3.2 ± 1.0^Aa^	3.3 ± 1.1^Ba^	3.1 ± 0.9^Ba^
Stickiness/60	3.2 ± 0.6^Ba^	3.3 ± 0.9^Aa^	3.4 ± 0.9^ABa^	3.3 ± 0.8^Ba^
Stickiness/90	3.0 ± 0.7^Ba^	3.1 ± 1.0^Aa^	3.2 ± 1.1^Ba^	3.1 ± 0.9^Ba^
General Acp/1	7.4 ± 0.5^Ac^	8.2 ± 0.8^Abc^	8.6 ± 1.0^Ab^	8.8 ± 0.4^Aa^
General Acp/30	6.9 ± 0.9^Ab^	7.9 ± 0.9^Aa^	8.3 ± 0.5^Aa^	8.4 ± 0.8^Aa^
General Acp/60	6.7 ± 0.5^Ac^	7.8 ± 0.6^Ab^	8.4 ± 0.5^Aab^	8.7 ± 0.7^Aa^
General Acp/90	7.2 ± 0.4^Ac^	8.2 ± 0.6^Ab^	8.7 ± 0.4^Aab^	8.8 ± 0.4^Aa^

*Note*: Data represent average values ± standard deviation. There is no significant difference between the results shown with the same capital letter in the same column for the same parameter (*p* > .05). There is no significant difference between the results shown with the same lowercase letter in the same line (*p* > .05). P: phycocyanin, 0, 0.1, 0.3, and 0.5: phycocyanin additive percentage, Acp: acceptability.

In product development studies, changes in formulation or adding a new ingredient can alter the taste and aroma perception of the product (Guichard, 2002; Luzardo‐Ocampo et al., 2021). For example, carotenoids are susceptible to isomerization and oxidation during processing and storage of foods and their oxidized compounds can cause off‐flavor and undesirable results (Eun et al., 2020; Sigurdson et al., 2017). As natural food colorants, anthocyanins can also cause off‐flavors in food products as they tend to deteriorate rapidly due to different factors, such as light, oxygen, enzymes, metals, presence of other oxidants, pH, and temperature (Mattioli et al., 2020). Likewise, it is stated that various microalgae‐based additives with coloring properties may affect the taste and flavor of the products (Grahl et al., 2018; Nunes et al., 2023). From this point of view, in the present study, the coloring of toffee‐type candy samples with different concentrations of phycocyanin did not cause any change in taste of the products (*p* > .05).

**FIGURE 3:**
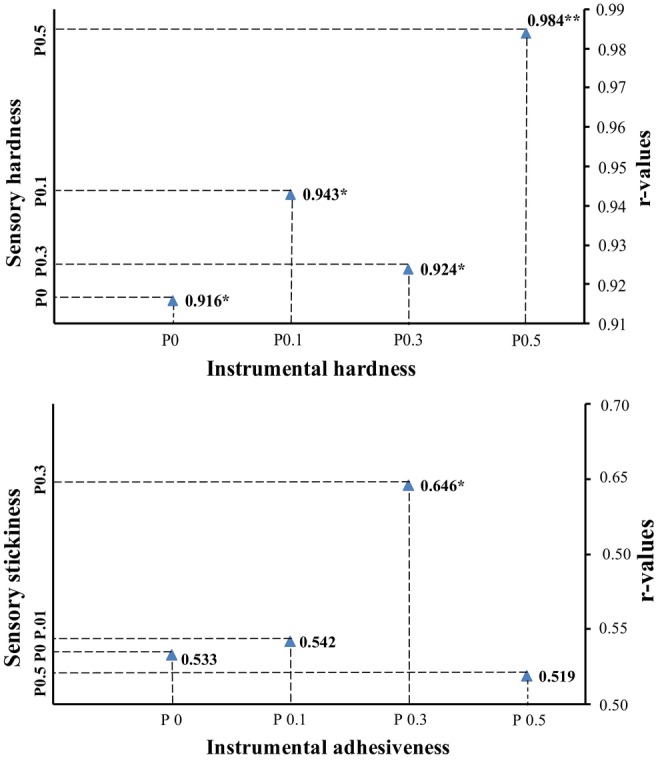
Evaluation of the sensory analysis scores and the texture analysis results obtained for 3 months as a whole with the Pearson correlation test of the sample groups stored at 20 ± 2°C. P, phycocyanin, 0, 0.1, 0.3, and 0.5: phycocyanin additive percentage. *Correlation is significant at the 0.05 level (one‐tailed). **Correlation is significant at the 0.01 level (one‐tailed).

Considering the sensory hardness and stickiness results, it was determined that phycocyanin addition did not have any negative effects on the scores (*p* > .05). The point that draws attention here is that there is an increase in the hardness scores of all groups depending on the storage time, and this is thought to be due to the moisture loss of the samples over time. Identifying possible correlations between sensory and instrumental analysis is essential for predicting consumer response (Li et al., 2022; Szczesniak, 1987). On the other hand, although both sensory and instrumental techniques have different advantages and disadvantages, the best way to use sensory and instrumental tests is to consider the correlations between the two measurements in terms of food quality control (Barrett et al., 2010; Singham et al., 2015). In this context, in the present study, sensory analysis results and instrumental texture parameters were evaluated with the Pearson correlation test, and possible correlations are summarized in Figure 3. According to the results, a very high correlation was found between the sensory and textural hardness results obtained during the storage period of each group. In this regard, it can be stated that instrumental analyses of product hardness in terms of toffee‐type candy products give consistent responses to the results of sensory evaluation. In addition, a moderate correlation was detected between sensory stickiness and instrumental adhesiveness results. Brenner and Nishinari (2014) stated that the correlation between instrumental adhesiveness and the perception of textural stickiness is expected to be higher for more sticky foods where the adhesiveness is the primary texture attribute. On the other hand, Jeltema et al. (2015) divided consumers into four groups according to their oral behavior during food consumption; crushers, chewers, smooshers, and suckers. Therefore, due to the differences in consumer oral behavior, it can be expected that the sample stickiness scores of the panelists do not fully meet the instrumental results in our study. To sum up, all the phycocyanin added samples were appreciated by the panelists with high scores, and there was no decrease in the general acceptability scores of the samples during storage (Table 5). Regarding all these results as a whole, the use of phycocyanin in toffee‐type soft candy production resulted in high consumption appeal.

## 4 CONCLUSION

In this study, toffee‐type candy samples were colored with different concentrations of phycocyanin and the main purpose of the study was to determine the stability of this compound at various storage temperatures. First, phycocyanin powder did not cause any change in the water activity of the samples on the first day, but a linear decrease was observed in the values depending on the storage time. The incorporation of phycocyanin altered the L*, a*, and b* values of the samples and the increase in the concentration of the pigment decreased the L* values and increased the blueness (‐b*) values of the samples. According to the results at different temperatures, it was determined that the Δ*E* values on the 90th day calculated for the temperatures of 4, 20, and 40°C varied between 0.16–0.40, 2.45–2.76, and 6.52–7.90 for the samples, respectively. Considering these results, it can be stated that phycocyanin‐enriched toffee‐type candy products will remain stable on market shelves at room temperature by preserving their natural blue color for a long time. In addition, the texture of the colored samples with different amounts of phycocyanin did not show a different structure from the control; however, changes were determined depending on the storage time, independent of the phycocyanin enrichment. The panelists appreciated all concentrations of phycocyanin and significant correlations were obtained between some parameters in sensory and texture analysis. In future studies, it will be useful to focus on the functional properties of phycocyanin added candy products in order to enrich the literature on this subject.

## AUTHOR CONTRIBUTIONS

**Ibrahim Palabiyik:** Conceptualization (equal); methodology (equal); supervision (equal); writing – review and editing (equal). **Recep Gunes:** Formal analysis (equal); methodology (equal); writing – original draft (equal); writing – review and editing (equal). **Sefik Kurultay:** Conceptualization (equal); writing – review and editing (equal).

## CONFLICT OF INTEREST STATEMENT

The authors declare no conflict of interest and no competing financial interest.

## ETHICS STATEMENT

The study was reviewed and approved by the Tekirdag Namik Kemal University and informed permission was acquired from each individual before their involvement in the study.

## Data Availability

The data that support the findings of this study are available from the corresponding author upon reasonable request.
